# Galactocele in a Male Infant with Transient Hyperprolactinaemia: An Extremely Rare Cause of Breast Enlargement in Children

**DOI:** 10.1155/2016/9487616

**Published:** 2016-09-26

**Authors:** C. T. Lau, K. K. Y. Wong, P. Tam

**Affiliations:** Department of Surgery, The University of Hong Kong, Queen Mary Hospital, Pokfulam, Hong Kong

## Abstract

Galactocele is a rare breast condition in infants. Here, we report a 16-month-old boy who developed progressive left breast enlargement. Ultrasonography and magnetic resonance imaging revealed a 4 cm cystic lesion at left breast. Hormonal assay showed transient hyperprolactinaemia with no known cause identified. Subsequently, galactocele was confirmed on histopathological examination after complete surgical excision. No recurrence was observed on regular follow-up.

## 1. Introduction

Galactocele is a benign breast condition defined as cystic dilatation of the mammary gland containing milk [1]. It is an uncommon condition and usually occurs in lactating woman [2]. This lesion is an exceedingly rare cause of breast enlargement in paediatric patients with only a handful of cases reported in the literature [3–6]. In this article, we presented another case of unilateral galactocele in a male infant with hyperprolactinaemia.

## 2. Case Presentation

**Figure 1:**
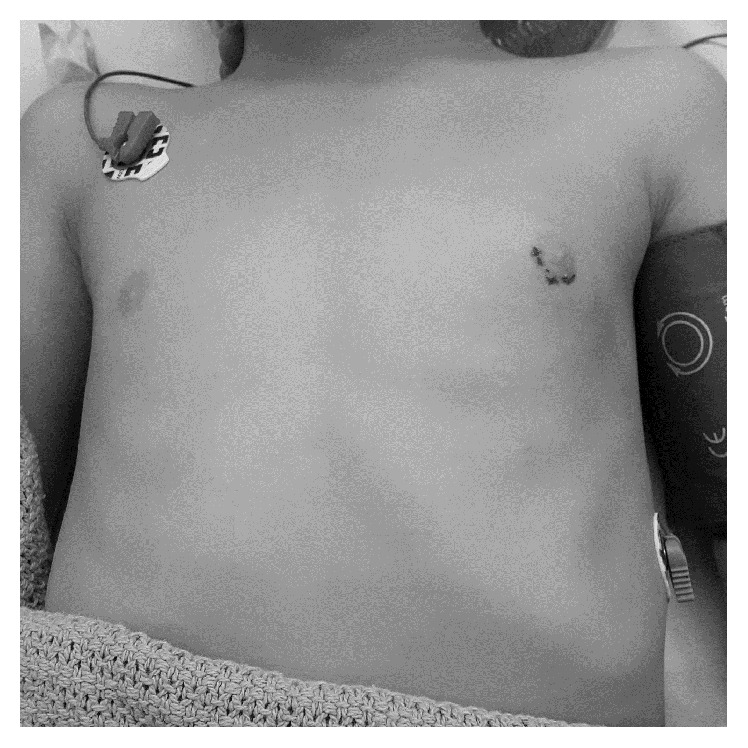
Preoperative photo showing left breast enlargement.

Our patient was a 16-month-old boy presented with a half-year history of left breast mass. The boy was born at full term by normal vaginal delivery with an uneventful pregnancy. He was all along healthy until left breast enlargement was noticed since 9 months of age. Initially, parents thought that the breast problem was transient and did not pay attention to it. However, the mass gradually increased in size over a course of 6 months which prompted the parents to seek medical attention. There was no history of trauma or infection of the left breast. No medication was taken by the mother during or after the pregnancy. The boy was exposed to neither any medication nor any hormonal products. There was no nipple discharge all along, and the family enjoyed good health with no specific history of breast disease. On physical examination, there was a painless mobile cystic mass just beneath the left nipple measuring 4 cm in diameter (Figure 1). The overlying nipple and skin were normal, and no nipple discharge was yielded upon gentle pressure. The right breast was normal. Examination of external genitalia revealed a normal male appearance with 4 cm penile length. Bilateral testes were descended with normal size, 1-2 mL in volume. The body length was 80 cm (25th to 50th percentile). There was no sign of puberty, hirsutism, or other abnormal pigmentation which signifies underlying endocrine problem.

**Figure 2:**
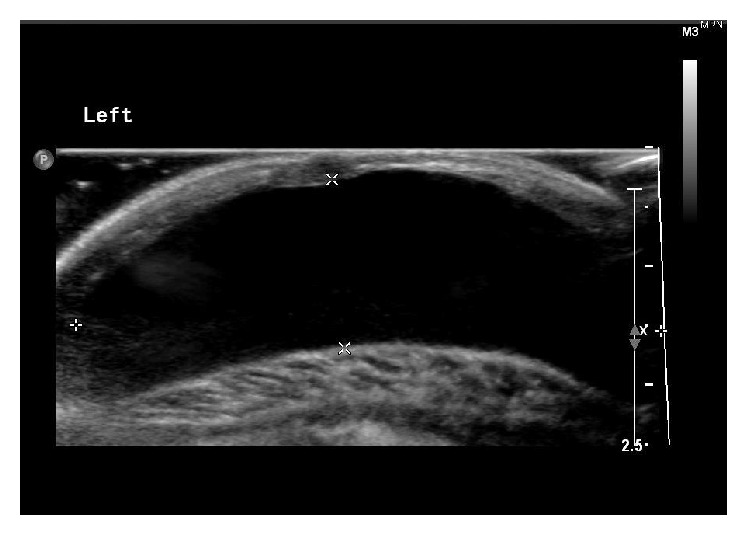
Ultrasonography of left breast showing a cystic lesion.

**Figure 3:**
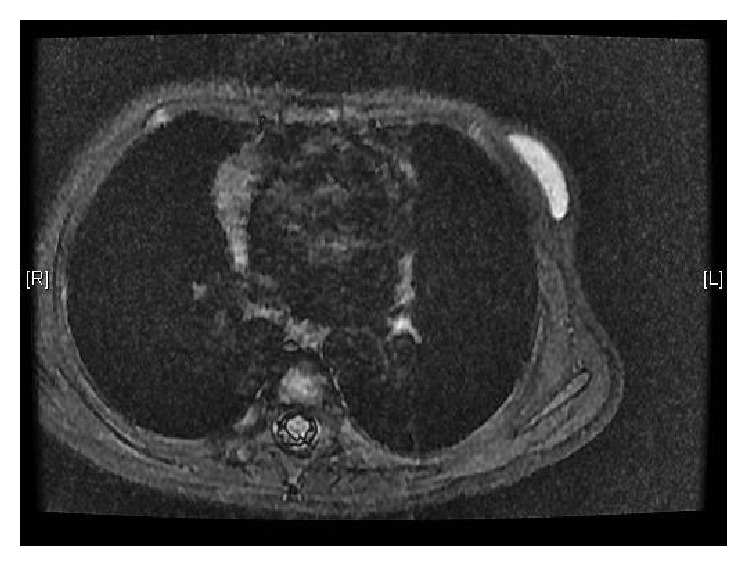
Magnetic resonance imaging showing a T1 hypertense lesion in left breast.

Ultrasonography of the left breast revealed a well circumscribed subareolar prepectoral cystic lesion, measuring 4.0 × 1.4 × 4.0 cm (Figure 2). Magnetic resonance imaging showed a 4.2 × 1.6 × 3.1 cm cystic lesion at left breast which is more hyperintense than muscle in T1 weighted sequence (Figure 3). There was slight rim enhancement after contrast injection. The right breast was normal in both ultrasound and magnetic resonance imaging.

Blood test showed high initial prolactin level at 549 mIU/L (normal < 500 mIU/L) without macroprolactinaemia, but it soon became normalized on a repeated test one month later. The rest of the endocrine profile revealed normal levels of luteinizing hormone < 0.5 IU/L (normal < 1 IU/L), follicle-stimulating hormone 0.89 IU/L (normal < 7 IU/L), estradiol 32 pmol/L (normal < 30 pmol/L), thyroid stimulating hormone 3.2 mIU/L (normal 0.35–4.78 mIU/L), and free thyroxine 16 pmol/L (normal 12–23 pmol/L). Other investigations including complete blood count, liver and renal function tests, and serum electrolytes were all unremarkable. A normal male karyotype of 46 XY was confirmed in chromosomal analysis. The result of bone age evaluation corresponded to the chronological age.

Needle aspiration was performed and yielded 5 mL whitish fluid. The fluid was positive for chyle. Biochemical tests of the fluid showed pH 7.5, triglycerides 39.2 mmol/L, cholesterol 4.5 mmol/L, protein 33 g/L, and glucose < 0.5 mmol/L. Slide review only showed presence of scanty lymphocytes with no malignant cell identified. Fluid culture was negative for bacterial growth.

**Figure 4:**
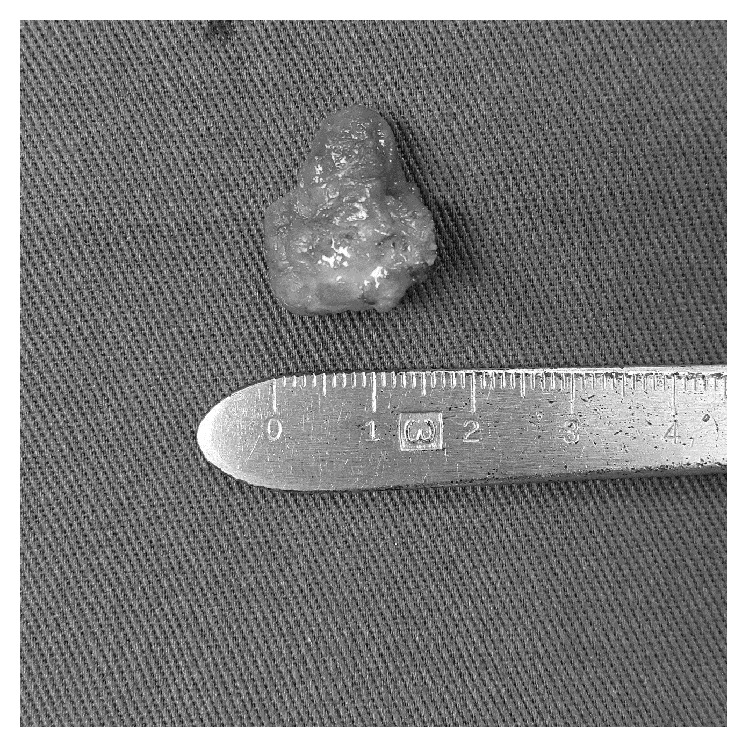
Specimen after excision.

The breast mass was followed up for one year with no sign of spontaneous regression and thus surgery was performed at 3 years old. The mass was excised in total with a semicircular infra-areolar incision uneventfully (Figure 1). Histopathological examination of the excised specimen revealed an irregularly shaped cystic tissue containing dilated ducts, which were lined by cuboidal epithelium with proteinaceous secretory material in the luminal space (Figure 4). There was no evidence of in situ or invasive malignancy and the diagnosis was compatible with galactocele. Patient recovered well from the operation. He had regular follow-up both clinically and sonographically every 3 months, and no recurrence was noticed one year after the operation. Other growth parameters remained steady on reassessment.

## 3. Discussion

Galactocele is most commonly found in young fertile women during or after breastfeeding [2]. It is an extremely rare cause of breast enlargement in infants and children. Interestingly, all infants reported with the diagnosis of galactocele were male. Up till now, only 29 cases have been reported in the literature [3–6].

The exact mechanism of galactocele formation in infants is still unknown, but in the majority of cases (22 cases) reported previously galactocele was not associated with any other abnormalities. Three other reported cases were associated with isolated congenital malformation: renal dysplasia, ventricular septal defect, and cleft lip, respectively. While some author suggested that the cause may be multifactorial [7], several specific factors had been postulated. These factors include a previous history of trauma or inflammation leading to breast cyst formation in the neonatal period, previous or present stimulation by prolactin, the presence of secretory breast epithelium, and ductal obstruction [8]. In the literature, there were only 4 galactocele patients noted to have hyperprolactinaemia; 2 of them were persistent and 2 of them transient. Hypothyroidism [9], presence of macroprolactin [10], celiac disease [11], and even physical examination [5] have been speculated as the cause of hyperprolactinaemia in children. Of particular interest, 2 of these patients with hyperprolactinaemia also had midline defect, one with cleft lip and the other one with cleft palate. This is compatible with the hypothesis that, in patient with midline defect, the tuberoinfundibular dopaminergic pathway may be impaired, which will lead to a decreased inhibition of prolactin secretion by the pituitary gland with resulting galactocele [12]. Our patient had transient hyperprolactinaemia as well, but it was not associated with nipple discharge or other congenital abnormalities. No specific cause had been identified in our patient.

Another point to note was the high normal estradiol level in our patient. The association between estradiol level and galactocele formation had not been previously described by other authors. Estradiol level tested using immunoassay can be falsely raised in the presence of other exogenous steroid compounds, including medication or dietary intake, in which our patient did not recall any history of these. There was no clinical or radiological sign of precocious puberty; in addition, the rise of estradiol level was only transient and other hormone levels were normal, making this diagnosis unlikely. Nonetheless, additional research should be warranted to fully investigate the relationship between estradiol and galactocele in the future.

Lymphatic malformation is the most important differential diagnosis in infants with cystic breast lesions because it is much more common. Even with the application of ultrasonography, the diagnosis can still be a challenge to radiologists. Magnetic resonance imaging certainly provides better soft tissue details with less operator dependency but has been performed in only 4 cases previously [3, 13, 14]. Needle aspiration is a simple and reliable diagnostic tool which confirms the diagnosis with aspiration of milky fluid. Other differential diagnoses include vascular malformation and gynaecomastia has to be thoroughly considered before operation [13]. Some authors suggested aspiration can be performed as the definitive therapeutic procedure alone but had been reported in 3 cases only. All other cases were treated by complete surgical excision with no recurrence reported so far [3].

In conclusion, galactocele is an extremely rare disease in infants and was found exclusively in male. We reported the 30th case of galactocele in infant published in English literature. Clinicians should not overlook this entity as a possible diagnosis and more research is needed to find out its underlying pathophysiology.
